# Early Invasive Strategy and In‐Hospital Survival Among Diabetics With Non‐ST‐Elevation Acute Coronary Syndromes: A Contemporary National Insight

**DOI:** 10.1161/JAHA.116.005369

**Published:** 2017-03-18

**Authors:** Ahmed N. Mahmoud, Islam Y. Elgendy, Hend Mansoor, Xuerong Wen, Mohammad K. Mojadidi, Anthony A. Bavry, R. David Anderson

**Affiliations:** ^1^ Division of Cardiovascular Medicine Department of Medicine University of Florida Gainesville FL; ^2^ Department of Pharmaceutical Outcomes and Policy University of Florida Gainesville FL; ^3^ College of Pharmacy University of Rhode Island Kingston RI; ^4^ Cardiology Section (111D) North Florida/South Georgia Veterans Health System Malcom Randall Veterans Administration Medical Center Medical Service Gainesville FL

**Keywords:** acute coronary syndrome, early invasive strategy, mortality, propensity score analysis, Catheter-Based Coronary and Valvular Interventions, Percutaneous Coronary Intervention, Revascularization, Mortality/Survival, Coronary Artery Disease

## Abstract

**Background:**

There are limited data on the merits of an early invasive strategy in diabetics with non‐ST‐elevation acute coronary syndrome, with unclear influence of this strategy on survival. The aim of this study was to evaluate the in‐hospital survival of diabetics with non‐ST‐elevation acute coronary syndrome treated with an early invasive strategy compared with an initial conservative strategy.

**Methods and Results:**

The National Inpatient Sample database, years 2012–2013, was queried for diabetics with a primary diagnosis of non‐ST‐elevation acute coronary syndrome defined as either non‐ST‐elevation myocardial infarction or unstable angina (unstable angina). An early invasive strategy was defined as coronary angiography±revascularization within 48 hours of admission. Propensity scores were used to assemble a cohort managed with either an early invasive or initial conservative strategy balanced on >50 baseline characteristics and hospital presentations. Incidence of in‐hospital mortality was compared in both groups. In a cohort of 363 500 diabetics with non‐ST‐elevation acute coronary syndrome, 164 740 (45.3%) were treated with an early invasive strategy. Propensity scoring matched 21 681 diabetics in both arms. Incidence of in‐hospital mortality was lower with an early invasive strategy in both the unadjusted (2.0% vs 4.8%; odds ratio [OR], 0.41; 95% CI, 0.39–0.42; *P*<0.0001) and propensity‐matched models (2.2% vs 3.8%; OR, 0.57; 95% CI, 0.50–0.63; *P*<0.0001). The benefit was observed across various subgroups, except for patients with unstable angina (*P*_interaction_=0.02).

**Conclusions:**

An early invasive strategy may be associated with a lower incidence of in‐hospital mortality in patients with diabetes. The benefit of this strategy appears to be superior in patients presenting with non‐ST‐elevation myocardial infarction compared with unstable angina.

## Introduction

Diabetes mellitus (DM) is a rapidly growing global health burden. In 2014, the prevalence of DM was estimated to be 422 million worldwide, doubling in frequency since 1980.^1^ In the United States, the prevalence of DM increased dramatically from 3.5% in 1990 to 8.3% in 2012.^2^ DM is a predominant risk factor for atherosclerotic coronary artery disease (CAD) and acute myocardial infarction (MI).^3^, ^4^ Diabetics have a higher incidence of multivessel CAD, accelerated atherosclerosis, atherosclerotic plaque rupture, and increased platelet activity, all of which increase the incidence of acute MI compared to nondiabetics.^4^ Additionally, DM is independently associated with a higher incidence of early and late mortality following a non‐ST‐elevation acute coronary syndrome (NSTE‐ACS).^5^, ^6^

There is currently a paucity of data on the benefit of an early invasive strategy in diabetic patients with NSTE‐ACS, given the limited number of diabetic patients enrolled in most randomized, clinical trials.^7^ Multiple observational studies and meta‐analyses of randomized trials demonstrate the benefit of an early invasive strategy in management of diabetic NSTE‐ACS patients, mainly through reduction of composite clinical event rates and MI.^8^, ^9^ As a direct consequence, the American College of Cardiology Foundation/American Heart Association (ACCF/AHA) Task Force recommends a routine invasive strategy (within 72 hours of hospitalization) for diabetics presenting with NSTE‐ACS.^10^ However, evidence from real‐life registry data indicates that diabetics are less frequently offered acute reperfusion therapy or acute revascularization compared to nondiabetics.^6^, ^11^, ^12^

To date, there is insufficient evidence to support a survival benefit of an early invasive strategy in diabetic patients with NSTE‐ACS. The aim of this study is to evaluate the effect of an early invasive strategy in NSTE‐ACS diabetic patients, with emphasis on survival, length of hospital stay, and cost.

## Methods

### Study Data Sources

The National Inpatient Sample (NIS) database (years 2012 and 2013) was used to collect data for the current study. Years 2012 and 2013 were chosen because those were the most recent NIS database releases at the time this study was conducted and thus would most closely reflect contemporary management of NSTE‐ACS. In 2012, the NIS was redesigned to include a random sample of patient discharges from all hospitals, rather than a random sample of hospitals retaining their discharges, which resulted in more‐precise national estimates. The NIS is the largest all‐payer database in the United States available for public use and sponsored by the Agency for Healthcare Research and Quality (AHRQ) as a part of the Healthcare Cost and Utilization Project.^13^ It represents a 20% stratified sample of all discharges from the US community hospitals. Each individual patient hospitalization is de‐identified and maintained as a specific entry. Data available in the NIS include 1 primary and 24 secondary diagnoses, 25 procedure diagnoses (in International Classification of Diseases, Ninth Edition, Clinical Modification [ICD‐9‐CM] coding format), patient demographic characteristics (eg, sex, age, race, and median household income), hospital characteristics (eg, ownership), expected payment source, total hospital charges, discharge status, length of hospital stay, as well as, severity and comorbidity measures. Discharge weights are also available for each patient's record, which could be used in national weighted estimates generation. This study was deemed to be exempt from the Institutional Review Board (IRB) because of the public nature of the NIS database, with the absence of any personal identifying information.

### Validation and Data Control

The NIS performs annual data quality assessments, to ensure the internal validly of its data. It had been previously compared with alternative databases, such as American Hospital Association Annual Survey Database, the National Hospital Discharge Survey from the National Center for Health Statistics, and the Med‐PAR inpatient database from Centers for Medicare and Medicaid Services, with comparable estimates to all of the previously stated databases.^14^

### Patient Population

All patients with a primary diagnosis code of non‐ST‐elevation myocardial infarction (NSTEMI) (ICD‐9‐CM code of 410.7x) or unstable angina (UA; ICD‐CM 9 code of 411.1) and secondary diagnosis of diabetes mellitus (according to the Elixhauser comorbidity software defined by the AHRQ) were included.^15^ The analysis was limited to patients with the primary diagnosis of NSTE‐ACS as it is usually considered the main reason for admission in the NIS database. This would allow the exclusion of patients with type 2 NSTEMI secondary to other concomitant diseases. An early invasive strategy group was defined as coronary angiography (ICD‐9‐CM codes of 88.55, 37.22, or 37.23) with or without revascularization, that is, percutaneous coronary intervention (PCI; ICD‐9‐CM codes of 00.66, 36.01, 36.02, 36.05, 36.06, and 36.07) or coronary artery bypass grafting (CABG; ICD‐9‐CM code 36.1x) with procedure time being within 48 hours of admission (ie, day 0 or 1). The remaining patients were defined as an initial conservative strategy group. This definition of an early invasive strategy in NSTE‐ACS has previously been used for other studies utilizing data from the NIS database.^16^, ^17^

### Study Outcomes

The main outcome of this study was in‐hospital mortality, referred to in the NIS database as the “DIED” variable. The main outcome was compared in both the early invasive and initial conservative strategy groups after adjusting for multiple patient and hospital characteristics. Other outcomes of interest were length of hospital stay, referred in the NIS database as “LOS” variable and total hospital charges, referenced as the “TOTCHG” variable. The total hospital charges represent the total amount billed by the hospital for service rather than the actual payment received.

### Patients and Hospital Characteristics

Data variables collected were patients' demographics, including age, sex, race, median household income by ZIP code, weekend versus weekday admission, and primary expected payer (Medicare, Medicaid, Private insurance, Uninsured, or Other) and the Elixhauser list of comorbidities. Other relevant diagnoses were also extracted by their corresponding ICD‐9‐CM codes, including acute ischemic stroke, intracranial hemorrhage, gastrointestinal bleeding, cardiogenic shock, family history of coronary artery disease (CAD), history of past MI, past PCI or CABG, past stroke or transient ischemic attack (TIA), CAD, carotid artery disease, smoking, dyslipidemia, atrial fibrillation, and dementia. Hospital characteristics, such as bed size (small, medium, and large), urban location, and teaching status, were also collected.^18^ A full list of ICD‐9‐CM codes for all variables collected in the current study is supplied in Table 1.

**Table 1:** International Classification of Diseases, Ninth Edition, Clinical Modification (ICD‐9‐CM) Codes of the Variables Included in the Propensity Score Matching^a^

Variable	ICD‐9‐CM Code
Acute ischemic stroke	433.01, 433.11, 433.21, 433.31, 433.81433.91, 434.01, 434.11, 434.91, 435.x, 436
Intracranial hemorrhage	430, 431, 432.x
Gastrointestinal bleeding	153
Cardiogenic shock	785.51
Family history of CAD	V17.3
Past MI	412
Past PCI	V45.82
Past CABG	V45.81
Past stroke/TIA	V12.54
CAD	414.00, 414.01, 414.02, 414.03, 414.04, 414.05, 414.06, 414.07
Carotid artery disease	433.10
Smoking history	V15.82, 305.1
Dyslipidemia	53
Atrial fibrillation	427.31
Dementia	290.xx, 294.1x, 294.2x, 294.8, 331.0–331.12, 331.82, 797

CABG indicates coronary artery bypass grafting; CAD, coronary artery disease; MI, myocardial infarction; PCI, percutaneous coronary intervention; TIA, transient ischemic attack.

a Other variables not reported in the table were collected using Elixhauser Comorbidity Software.^15^

### Statistical Analysis

Weighted national estimates were calculated using the discharge weights supplied by the NIS. Frequencies and percentages were used for estimation of categorical patient and hospital characteristics and means with SD or medians, with 25th to 75th percentile ranges, for continuous ones. Categorical variables were compared by Pearson's chi‐square test, and an independent‐sample Student *t* test was used for means comparison in both groups. Medians were compared using Mood's median test. A multivariable logistic regression model was constructed using all previously stated patient and hospital characteristics as independent variables, with early invasive strategy being the dependent variable. The resultant individually matched probability score was then used for propensity score matching of 2 similar groups (an early invasive strategy vs initial conservative strategy) with 1:1 ratio and match tolerance of 0.01. A clinically significant difference between both groups was considered present if the absolute difference in frequency or means was >5% postmatching. The propensity‐matched cohort of patients was then used to compare the incidence of different outcomes of interest. The odds ratio (OR) of in‐hospital mortality was calculated in the propensity‐matched data using a binary conditional logistic regression model.

A subgroup analysis was conducted comparing the OR of in‐hospital mortality according to the use of an early invasive strategy across various predefined subsets, including NSTEMI versus UA, history of congestive heart failure (CHF), presence of cardiogenic shock, males versus females, and past history of MI, CAD, or revascularization (PCI or CABG). A secondary propensity adjusted multivariable logistic regression analysis was also calculated for in‐hospital mortality with all previously stated variables along with the use of early invasive strategy and the propensity score as independent variables. The regression was performed by a backward step‐wise approach with a cut‐off level of 0.05 for entry and 0.1 for removal, followed by calculation of an adjusted OR for in‐hospital mortality.

To take into account the possibility of an immortality time bias, a sensitivity analysis was conducted after exclusion of NSTE‐ACS patients who had a length of hospital stay less than 48 hours (ie, day 0 and 1) in the propensity‐matched cohort. Immortal time bias refers to a period of follow‐up during which, by design, the study outcome cannot occur.^19^ Limiting the analysis to patients, who had a length of hospital stay more than 48 hours, was an indirect method of analyzing patients who survived for at least 48 hours, as the time of death was not a reported variable in the NIS database.

Finally, another propensity score analysis was conducted using a tighter match tolerance of 10e^−5^ to confirm the findings of the primary analysis, because a statistically significant difference in the frequency of some categorical variables was detected in the primary analysis. All statistical analyses were performed using IBM SPSS Statistics software (version 23.0; IBM Corp., Armonk, NY) with 2‐sided *P* value of <0.05 for statistical significance assessment for all analyses and OR with 95% CI as a measure of effect size reported by logistic regression.

## Results

### Baseline Characteristics

We identified 363 500 diabetic patients who were admitted in the United States with a primary diagnosis of NSTE‐ACS (mean age, 68.1±12.6 years; 42.9% female; 64.5% being white; 65.5% covered by Medicare) during the years 2012–2013. Most of the patients were hypertensive (82.8%) and had a past history of CAD (77.9%). A total of 229 435 patients (63.1%) underwent an invasive strategy (early or delayed) during their admission (coronary angiography, PCI, or CABG). Of the total patient population, 164 740 patients (45.3%) had an early invasive strategy performed during their hospital stay. Total incidence of in‐hospital mortality was 12 925 patients (3.9%). Patient and hospital characteristics of the included patients are summarized in Table 2.

**Table 2:** Patient and Hospital Characteristics of Both the Unmatched and Propensity‐Score–Matched Cohort of Patients

Variable	Unmatched Patients		*P* Value	Propensity‐Score Matched		*P* Value^a^
	Early Invasive (%)	Initial Conservative (%)		Early Invasive (%)	Initial Conservative (%)	
Total number of patients	164 740 (100)	198 760 (100)		21 681 (100)	21 681 (100)	
Primary diagnosis						
NSTEMI^b^	161 020 (98)	185 055 (93)	<0.0001	21 130 (98)	20 247 (93)	<0.0001
UA^b^	3720 (2)	13 705 (7)	<0.0001	550 (3)	1434 (7)	<0.0001
Patient demographics						
Age, mean y (SD)	65 (12)	71 (13)	<0.0001	67 (11)	68 (13)	0.09
Female sex	64 130 (39)	91 945 (46)	<0.0001	9044 (42)	9176 (42)	0.20
Race			<0.0001			<0.0001^a^
White	109 040 (70)	128 515 (69)		15 063 (70)	14 983 (69)	
Black	20 000 (13)	26 500 (14)		2768 (13)	3163 (15)	
Hispanic	16 295 (10)	20 015 (11)		2227 (10)	2093 (10)	
Asian or Pacific Islander	4400 (3)	5660 (3)		606 (3)	577 (3)	
Other	6110 (4)	5655 (3)		868 (4)	710 (3)	
Primary expected payer			<0.0001			0.02^a^
Medicare	95 625 (58)	142 420 (72)		14 157 (65)	14 262 (66)	
Medicaid	12 845 (8)	14 590 (7)		1477 (7)	1798 (8)	
Private insurance	39 630 (24)	28 845 (14)		4327 (20)	3902 (18)	
Uninsured	10 160 (6)	7390 (3)		1037 (5)	1075 (5)	
Other	5110 (3)	4565 (2)		581 (3)	554 (3)	
Weekend admission	33 325 (20)	59 330 (30)	<0.0001	5279 (24)	5379 (25)	0.27
Household income (median)			<0.0001			<0.0001^a^
0 to 25th percentile	53 420 (33)	66 370 (34)		7384 (34)	7280 (34)	
26 to 50th percentile	43 660 (27)	51 265 (26)		5821 (27)	5668 (26)	
51 to 75th percentile	36 995 (23)	43 555 (23)		4895 (23)	4921 (23)	
76 to 100th percentile	27 175 (17)	32 760 (17)		3581 (17)	3812 (18)	
Patient characteristics						
Smoking	54 065 (33)	44 355 (22)	<0.0001	6067 (28)	5901 (27)	0.08
Dyslipidemia	125 075 (76)	131 195 (66)	<0.0001	15 786 (73)	15 666 (72)	0.20
Obesity	45 060 (27)	43 055 (21)	<0.0001	5449 (25)	5369 (25)	0.38
Known history of CAD	146 755 (89)	136 395 (69)	<0.0001	18 399 (85)	18 493 (85)	0.21
Family history of CAD	13 100 (8)	7365 (4)	<0.0001	1148 (5)	1115 (5)	0.48
Past MI	20 705 (13)	25 895 (13)	<0.0001	2922 (14)	3011 (14)	0.21
Past PCI	137 975 (84)	172 170 (87)	<0.0001	3519 (16)	3548 (16)	0.71
Past CABG	12 640 (8)	24 895 (13)	<0.0001	2178 (10)	2269 (11)	0.15
Past stroke or TIA	9150 (6)	13 390 (7)	<0.0001	1316 (6)	1321 (6)	0.92
Carotid artery disease	4080 (3)	4750 (2)	0.09	573 (3)	575 (3)	0.95
Peripheral vascular disease	25 335 (15)	36 090 (18)	<0.0001	3819 (18)	3817 (18)	0.98
Pulmonary circulation disease	145 (<1)	275 (<1)	<0.0001	21 (<1)	29 (<1)	0.26
Dementia	3000 (2)	15 350 (8)	<0.0001	562 (3)	508 (2)	0.10
Atrial fibrillation	22 725 (14)	39 970 (20)	<0.0001	3536 (16)	3578 (17)	0.59
Alcohol abuse	3525 (2)	3830 (2)	<0.0001	454 (2)	434 (2)	0.50
Deficiency anemia	32 325 (20)	55 310 (28)	<0.0001	5062 (23)	5101 (24)	0.66
Collagen vascular disease	3505 (2)	4410 (2)	0.06	480 (2)	471 (2)	0.79
Chronic blood loss anemia	1295 (1)	2085 (1)	<0.0001	197 (1)	200 (1)	0.88
Congestive heart failure	690 (<1)	2005 (1)	<0.0001	124 (<1)	145 (<1)	0.20
Valvular disease	195 (<1)	615 (<1)	<0.0001	36 (<1)	47 (<1)	0.23
Chronic pulmonary disease	35 540 (22)	52 430 (26)	<0.0001	5252 (24)	5322 (25)	0.43
Coagulopathy	9480 (6)	11 610 (6)	0.27	1313 (6)	1280 (6)	0.50
Liver disease	2895 (2)	4385 (2)	<0.0001	420 (2)	416 (2)	0.89
Renal disease (chronic)	41 815 (25)	78 900 (40)	<0.0001	6967 (32)	7005 (32)	0.70
Electrolytes abnormalities	34 070 (21)	56 890 (29)	<0.0001	5170 (24)	5203 (24)	0.71
AIDS	120 (<1)	175 (<1)	0.12	16 (<1)	14 (<1)	0.72
Drug abuse	3050 (2)	3425 (2)	<0.0001	392 (2)	394 (2)	0.94
Depression	14 495 (9)	19 320 (10)	<0.0001	1971 (9)	2040 (9)	0.25
Hypertension	138 115 (84)	162 920 (82)	<0.0001	18 088 (83)	18 086 (83)	0.98
Hypothyroidism	19 215 (12)	29 180 (15)	<0.0001	2801 (13)	2819 (13)	0.80
Lymphoma	705 (<1)	1240 (0.6)	<0.0001	110 (<1)	114 (<1)	0.79
Metastatic cancer	625 (<1)	2050 (1)	<0.0001	114 (<1)	106 (<1)	0.59
Solid tumor without metastasis	1720 (1)	3480 (2)	<0.0001	289 (1)	295 (1)	0.80
Other neurological disorder	7385 (5)	16 065 (8)	<0.0001	1142 (5)	1192 (6)	0.29
Paralysis	2735 (2)	5445 (3)	<0.0001	431 (2)	457 (2)	0.38
Psychoses	4040 (3)	6860 (4)	<0.0001	622 (3)	597 (3)	0.47
Peptic ulcer (nonbleeding)	35 (<1)	35 (<1)	0.43	4 (<1)	2 (<1)	0.41
Weight loss	3330 (2)	6725 (3)	<0.0001	542 (3)	518 (2)	0.46
Cardiogenic shock	6100 (4)	5220 (3)	<0.0001	678 (3)	654 (3)	0.50
Intracranial hemorrhage	150 (<1)	255 (<1)	<0.0001	21 (<1)	24 (<1)	0.66
Acute ischemic stroke	2315 (1)	3185 (2)	<0.0001	328 (2)	337 (2)	0.73
Gastrointestinal bleeding	2545 (2)	5530 (3)	<0.0001	433 (2)	455 (2)	0.46
Invasive procedure						
PCI^b^	159 585 (97)	59 810 (30)	<0.0001	10 415 (48)	3862 (18)	<0.0001
CABG^b^	25 650 (16)	11 760 (6)	<0.0001	3037 (14)	1820 (8)	<0.0001
Hospital characteristics						
Hospital bed size			<0.0001			0.05
Small	13 150 (8)	26 530 (13)		2062 (10)	2203 (10)	
Medium	40 040 (24)	54 740 (28)		5568 (26)	5553 (26)	
Large	111 550 (68)	117 490 (59)		14 051 (65)	13 925 (64)	
Hospital location			<0.0001			0.14
Urban teaching	92 320 (56)	91 430 (46)		11 133 (51)	11 532 (53)	
Urban nonteaching	61 130 (37)	81 580 (41)		8811 (41)	8213 (38)	
Rural	11 290 (7)	25 750 (13)		1737 (8)	1936 (9)	
In‐hospital mortality	3320 (2.0)	9605 (4.8)	<0.0001	475 (2.2)	826 (3.8)	<0.0001

All percentages are approximated to the nearest integer. AIDS indicates acquired immune deficiency syndrome; CABG, coronary artery bypass graft surgery; CAD, coronary artery disease; NSTEMI, non‐ST‐elevation myocardial infarction; MI, myocardial infarction; PCI, percutaneous coronary intervention; TIA, transient ischemic attack; UA, unstable angina.

a Although *P* value is significant the difference in % between both arms was <5% and thus deemed clinically nonsignificant.

b Variables were not included in the propensity score matching.

Compared with an initial conservative strategy, patients undergoing an early invasive strategy were younger (65.2 [SD=11.8] vs 70.6 [SD=12.8] years; *P*<0.0001), less frequently female (38.9% vs 46.3%; *P*<0.0001), and had fewer comorbidities (Table 2). The use of an early invasive strategy was more common in large‐bed‐size hospitals and in urban teaching hospitals (Table 2). Patients carrying a diagnosis of UA were less likely to undergo an early invasive strategy compared to those with NSTEMI (21.3% vs 45.3%; *P*<0.0001). The frequency of revascularization was higher in the early invasive strategy group by both PCI (96.9% vs 30.1%; *P*<0.0001) and CABG (15.6% vs 5.9%; *P*<0.0001) (Table 2). Nonetheless, all patients in the initial conservative strategy group who had a coronary angiography performed had a revascularization procedure (i.e. PCI and/or CABG) as well.

Propensity score matching yielded 21 681 patients in both groups with a similar distribution of patient and hospital characteristics between the 2 groups of interest, except for a few categorical variables such as expected primary payer (*P*<0.0001), race (*P*<0.0001), median household income (*P*=0.02), and hospital location (*P*<0.0001). However, all variables had absolute frequency differences less than 5% and thus deemed clinically insignificant (Table 2). To confirm our results, a secondary propensity score analysis was constructed with a tighter match tolerance of 10e^−5^ that yielded 12 363 patients in the early invasive strategy group and 12 367 in the initial conservative approach group, matched in all the formerly stated categorical variables (Table S1).

### In‐Hospital Mortality

**Figure 1:**
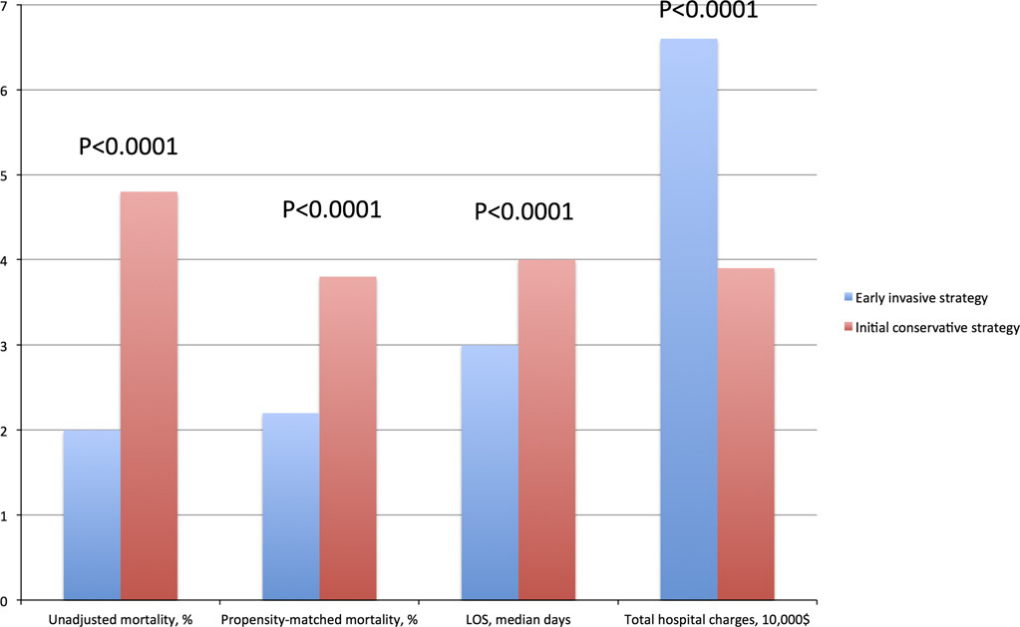
A cluster column graph comparing all outcomes of interest between an early invasive and an initial conservative strategy. LOS, length of hospital stay; $, US dollars. Both length of hospital stay and total hospital charges were derived from the propensity‐matched cohort of patients.

The incidence of in‐hospital mortality was lower with an early invasive compared with an initial conservative strategy in the unadjusted cohort (2.0% vs 4.8%; OR, 0.41; 95% CI, 0.39–0.42; *P*<0.0001). This benefit was maintained in the propensity‐matched cohort (2.2% vs 3.8%; OR, 0.57; 95% CI, 0.50–0.63; *P*<0.0001) and in the propensity‐adjusted multivariable logistic regression analysis (OR, 0.54; 95% CI, 0.52–0.57; *P*<0.0001; Figure 1). The incidence of in‐hospital mortality also was lower with an early invasive strategy in the secondary post‐hoc analysis using a tighter match tolerance (2.5% vs 3.7%; OR, 0.65; 95% CI, 0.56–0.75; *P*<0.0001) and in the sensitivity analysis after excluding the patients with length of hospital stay less than 48 hours in the propensity‐matched cohort (2.1 vs 3.3; OR, 0.63; 95% CI, 0.56–0.72; *P*<0.0001).

**Figure 2:**
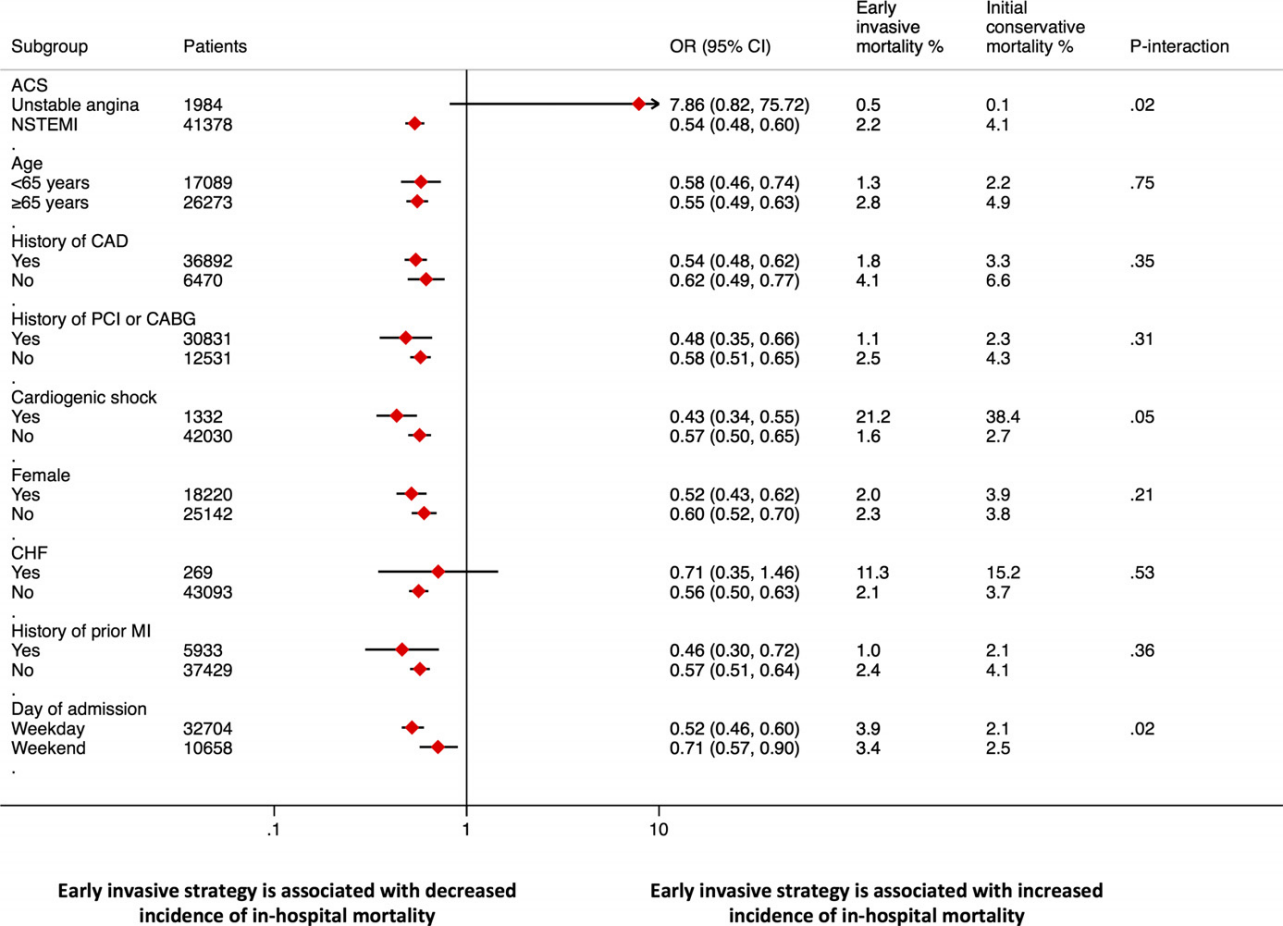
Forest plot representing subgroup analysis of in‐hospital mortality according to various patient risk factors. ACS indicates acute coronary syndrome; CABG, coronary artery bypass graft; CAD, coronary artery disease; CHF, congestive heart failure; MI, myocardial infarction; NSTEMI, non‐ST‐elevation myocardial infarction; OR, odds ratio; PCI, percutaneous coronary intervention.

On subgroup analysis, the benefit of an early invasive strategy was demonstrated among a wide range of prespecified subgroups except in patients with UA, where there was no apparent evidence of survival benefit with an early invasive strategy (0.5% vs 0.1%; OR, 7.86; 95% CI, 0.82–75.72; *P*=0.07), with evidence of heterogeneity when compared to NSTEMI patients (*P*_interaction_=0.02; Figure 2).

### Other Outcomes

An early invasive strategy was associated with a shorter length of hospital stay compared with an initial conservative strategy with a median of 3 (2–7) versus 4 days (2–7), respectively (*P*<0.0001), and higher total hospital charges with a median of 66 042 US dollars ($40315–112895) versus 39 265$ (19 193–77 832$), respectively (*P*<0.0001; Figure 1).

## Discussion

In the current propensity‐matched analysis of contemporary real‐life data, an early invasive strategy was associated with an increased in‐hospital survival in NSTE‐ACS patients with concomitant DM. These results support the 2014 ACCF/AHA guideline recommendations for an early invasive strategy in diabetics, especially those with high‐risk features (eg, NSTEMI and cardiogenic shock).^10^ Meanwhile, the use of this strategy in lower risk patients, such as those with UA, may not be associated with improved survival.

The survival benefit of an early invasive strategy in the NSTE‐ACS population remains a matter of ongoing debate.^20^, ^21^, ^22^ Whereas none of the landmark trials comparing an early invasive with an initial conservative strategy illustrated a statistically significant reduction in mortality, these trials were not statistically powered to answer that question.^21^, ^22^, ^23^, ^24^ We calculated the minimal sample size required by a randomized trial to detect the difference in proportions illustrated in our study and found that the sample size of almost 3500 patients would be necessary to obtain the same results,^25^, ^26^ which is more than double the number of diabetic NSTE‐ACS patients included in all previous trials combined.^9^ Although our results suggest an association between an early invasive strategy and improved survival in diabetics with NSTE‐ACS, this does not establish causality, given the retrospective nature of the data; thus, larger randomized, clinical trials powered for differentiation between short‐ and long‐term mortality are necessary to confirm our findings.

Earlier meta‐analyses of randomized trials have demonstrated a possible reduction in all‐cause mortality following an early invasive strategy, with a mean follow‐up of 1.5 to 2 years after the initial event.^20^, ^22^ However, subsequent meta‐analyses failed to replicate the same results.^7^, ^27^ A meta‐analysis of 9904 patients with NSTE‐ACS from 9 randomized trials investigated the benefit of an early invasive strategy use in diabetics (17% of the total population) compared to nondiabetics.^9^ Although the study did not show any added mortality benefit with an early invasive strategy, it showed a reduction in the rate of nonfatal MI.^9^

Interestingly, the benefit of an early invasive strategy was least evident in the subgroup of diabetics diagnosed primarily with UA. This is consistent with previous evidence showing a lack of benefit of an early invasive strategy in low‐risk NSTE‐ACS patients with low troponin levels^28^ and supports choosing an initial conservative strategy as a valid option in this subset of patients. Another finding was the infrequent use of an early invasive strategy in high‐risk diabetic patients admitted with NSTE‐ACS and cardiogenic shock. Only, 54% of the patients with cardiogenic shock underwent an early invasive strategy, however, those patients were the ones who benefited the most from this strategy with the lowest odds of in‐hospital mortality compared with both conservatively managed cardiogenic shock patients and noncardiogenic shock patients managed by an early invasive strategy.

A significant effect modification influenced by the admission day was evident, with less benefit noted with an early invasive strategy, if the patients were admitted during weekends compared with weekdays (*P*_interaction_=0.02). Although the exact reasons for such differences are not entirely clear, it may be attributed to lack of a readily available catheterization staff together with some human factors, such as sleep deprivation and fatigue as observed in previous studies.^29^ Despite this, the incidence of in‐hospital mortality remained significantly lower with an early invasive strategy when compared with an initial conservative strategy during the weekends (OR=0.71; 95% CI, 0.57–0.90; *P*<0.0001).

Even though a routine invasive strategy is recommended for diabetics by major cardiovascular guideline committees,^10^, ^30^ national registries worldwide have illustrated that this strategy is still underutilized.^6^, ^10^ Although the exact explanation for this finding is unclear, our analysis indicates that an early invasive strategy was used more frequently in younger patients with less comorbidity at baseline, which is usually not the case in most diabetics at the time of NSTE‐ACS presentation.

Although the definition of timing for an early invasive strategy (ie, within 48 hours) appears to be somewhat different in the current study compared with the ACC/AHA guidelines, the same definition was adopted by the pivotal trials comparing an early invasive strategy to an initial conservative strategy, such as Treat Angina with Aggrastat and Determine Cost of Therapy with an Invasive or Conservative Strategy—Thrombolysis in Myocardial Infarction 18(TACTICS‐TIMI 18; 4–48 hours),^31^ Invasive versus Conservative Treatment in Unstable Coronary Syndromes (ICTUS; 24–48 hours)^21^ and FRagmin and Fast Revascularisation during InStability in Coronary artery disease II (FRISC II; 24–48 hours)^23^ trials. A procedure time of 24 hours was used mainly to define an early invasive strategy in trials comparing early with delayed invasive strategy.^32^ Thus, we preferred to use the 48‐hour definition of an early invasive strategy to be consistent with previous trials comparing early invasive to initial conservative strategies in NSTE‐ACS patients.

To our knowledge, this study represents the largest contemporary analysis comparing an early invasive with an initial conservative strategy in diabetics with NSTE‐ACS. Although the NIS is an administrative database, the large sample size of patients that allowed adequate power to assess the main outcome of interest, together with the diverse demographics of the included patients, large number of patients' and hospital characteristics, and various in‐hospital complications recorded, made this database an excellent source to explore our question of interest. Multiple similar trend and outcome studies have been published using the NIS database, which validates its reliability in addressing various practice issues.^14^, ^16^, ^17^, ^33^ Despite these strengths, the current study has some limitations.

First, although we adjusted for over 50 independent variables in the propensity‐matched analysis, the current study is retrospective in nature and is subject to biases attributed to unmeasured confounders. Second, the inherent limitation of the NIS database, such as error in coding or misdiagnosis, may have occurred; however, given the large patient sample, we believe that such errors would be limited and should not affect the integrity of our results. Also, the NIS is an administrative database that relies mainly on ICD‐9 codes rather than clinically adjudicated outcomes or diagnoses. Third, because of the administrative nature of the NIS database, multiple clinical data were not accounted for, such as medications administered (eg, antiplatelet and ‐coagulant therapy), laboratory (eg, troponins and brain natriuretic peptide levels), and imaging (eg, echocardiography) results. This limited the ability to assess the merits of an early invasive strategy in various risk subgroups (eg, according to the Thrombolysis in Myocardial Infarction [TIMI] score). We attempted to assess the impact of past CAD and past revascularization history in our subgroup analysis as an indirect assessment of patients with higher TIMI score, and there was no evidence of effect modification by either. Lack of data regarding various types of medications administered is an important limitation, given that the inability to administer certain medications (eg, anticoagulant and ‐platelet agents) that are known to improve survival in NSTE‐ACS, because of the patients' underlying comorbidities, might have affected the choice of initial treatment strategy. Fourth, the individual subgroup analysis comparisons were not propensity matched, and thus the possibility of unmeasured bias cannot be excluded. Finally, given the nature of NIS data, we were limited to in‐hospital mortality and could not compare both strategies with regard to long‐term outcomes.

## Conclusions

In this large propensity score matched analysis, an early invasive strategy was found to be associated with improved in‐hospital survival in diabetic patients, especially those with high‐risk features, such as NSTEMI or cardiogenic shock. The use of an early invasive strategy in diabetics with UA does not appear to be beneficial with a possible signal of harm, and thus an initial conservative strategy may be a safer approach for these patients. Our findings should be confirmed with future trials that are powered for detection of short‐ and long‐term survival benefit of an early invasive strategy in diabetics with NSTE‐ACS.

## Sources of Funding

Publication of this article was funded in part by the University of Florida Open Access Publishing Fund.

## Disclosures

Dr Anderson is a consultant for Biosense Webster, a Johnson & Johnson Company. Dr Bavry received an honorarium from American College of Cardiology. All other authors have no potential conflicts of interest to disclose.

## Supporting information


**Table S1.** Patient and Hospital Characteristics of the Post‐Hoc Propensity‐Matched Cohort of Patients With Lower Match Tolerance
