# Supplementary material for: Early Invasive Strategy and In‐Hospital Survival Among Diabetics With Non‐ST‐Elevation Acute Coronary Syndromes: A Contemporary National Insight
Source: J Am Heart Assoc. 2017 Mar 18;6(3):e005369. doi: 10.1161/JAHA.116.005369 (PMC5524040; doi:10.1161/JAHA.116.005369)
Supplement: Supplementary file 1 — Table S1. Patient and Hospital Characteristics of the Post‐Hoc Propensity‐Matched Cohort of Patients With Lower Match Tolerance [file JAH3-6-e005369-s001.pdf]

SUPPLEMENTAL MATERIAL

Table S1. Patient and hospital characteristics of the post-hoc propensity matched cohort of patients with lower match tolerance.

Variable (%)	Propensity-score matched		P-value
	Early invasive (%)	Initial conservative (%)	
Number of patients	12,363(100)	12,367(100)	
**Patient demographics**			
Age, mean years (SD)	67.6(11)	67.7(12)	0.47
Female sex	5,122(41)	5,271(41)	0.06
Race			0.60
White	8,523(69)	8,543(69)	
Black	1,652(14)	1,690(13)	
Hispanic	1,321(11)	1,284(10)	
Asian or Pacific Islander	350(3)	333(3)	
Other	427(4)	419(3)	
Primary expected payer			0.13
Medicare	8,076(65)	8,173(66)	
Medicaid	960(8)	985(8)	
Private insurance	2,278(18)	2,237(18)	
Uninsured	662(5)	586(5)	
Other	326(3)	333(3)	
Weekend admission	3,064(25)	3,026(25)	0.58
Household income (median)			0.66
0-25th percentile	4,229(34)	4,229(34)	
26-50th percentile	3,248(26)	3,239(26)	
51-75th percentile	2,812(23)	2,761(22)	
76-100th percentile	2,074(17)	2,138(17)	
**Patient characteristics**			
Smoking	3,450(28)	3,366(27)	0.23
Dyslipidemia	8,919(72)	8,905(72)	0.81
Obesity	3,182(26)	3,103(25)	0.24
Known history of CAD	10,365(84)	10,527(85)	0.01
Family history of CAD	631(5)	624(5)	0.84
Prior myocardial infarction	1,668(14)	1,719(14)	0.57
Prior PCI	2,021(16)	2,034(16)	0.83
Prior CABG	1,251(10)	1,251(10)	0.99
Prior stroke or TIA	750(6)	730(6)	0.73
Carotid artery disease	325(3)	328(3)	0.91
Peripheral vascular disease	2,161(18)	2,231(18)	0.20
Pulmonary circulation disease	15(<1)	13(<1)	0.71
Dementia	353(3)	304(3)	0.05
Atrial fibrillation	2,027(16)	2,034(16)	0.91
Alcohol abuse	259(2)	260(2)	0.97
Deficiency anemia	2,936(24)	2,976(24)	0.56
Collagen vascular disease	254(2)	271(2)	0.46
Chronic blood loss anemia	116(1)	126(1)	0.52
Congestive heart failure	68(<1)	81(<1)	0.29
Valvular disease	15(<1)	20(<1)	0.40
Chronic pulmonary disease	3,013(24)	3,044(25)	0.66
Coagulopathy	781(6)	711(6)	0.43

Liver disease	236(2)	243(2)	0.75
Renal disease (chronic)	4,006(32)	4,044(33)	0.62
Electrolytes abnormalities	3,006(24)	3,030(25)	0.73
AIDS	11(<1)	9(<1)	0.65
Drug abuse	232(2)	225(2)	0.74
Depression	1,156(9)	1,154(9)	0.96
Hypertension	10,373(84)	10,290(83)	0.14
Hypothyroidism	1,579(13)	1,618(13)	0.47
Lymphoma	60(<1)	67(<1)	0.54
Metastatic cancer	60(<1)	74(<1)	0.23
Solid tumor without metastasis	165(1)	148(1)	0.33
Other neurological disorder	698(6)	679(6)	0.59
Paralysis	236(2)	250(2)	0.52
Psychoses	359(3)	334(3)	0.33
Peptic ulcer (non-bleeding)	1(<1)	1(<1)	1.00
Weight loss	317(3)	327(3)	0.69
Cardiogenic shock	400(3)	374(3)	0.34
Intra-cranial hemorrhage	9(<1)	15(<1)	0.22
Acute ischemic stroke	194(2)	182(2)	0.53
Gastrointestinal bleeding	245(2)	238(2)	0.75
**Hospital characteristics**			
Hospital bed size			0.25
Small	1,161(9)	1,238(10)	
Medium	3,178(26)	3,176(26)	
Large	8,024(65)	7,953(64)	
Hospital location			0.21
Urban teaching	6,309(49)	6,453(50)	
Urban non-teaching	5,033(41)	4,877(39)	
Rural	1,021(8)	1,037(8)	
**In-hospital mortality**	303(2.5)	462(3.7)	<0.0001

All percentages are approximated to the nearest integer.
CAD= coronary artery disease, PCI= percutaneous coronary intervention, CABG= coronary artery bypass graft surgery, TIA= transient ischemic attack, AIDS= acquired immune deficiency syndrome.
